# The Pioneer of the Sanatorium Idea

**Published:** 1916-07-01

**Authors:** 


### THE PIONEER OF THE SANATORIUM IDEA.

Dr. George Bodington has been justly regarded as the pioneer in modern times of the treatment of consumption in the open air, but it is often forgotten that he is entitled to be credited with something more than this in connection with the therapeutics of this disease. Not only did he set his face against the shutting up of consumptive patients in close and overheated rooms, but he inveighed against a prevalent practice which was probably equally harmful to these patients. This was the exhibition in often excessive doses of tartarised antimony, a remedy which we can well believe "materially assisted the disease by destroying the powers of nutrition, the muscular power, and the functions of the skin." This "antiphlogistic" treatment, as it was called, did nothing to aid the patient, but rather impaired what resistance he possessed. It is no wonder, then, that physicians regarded consumption as a hopeless disease to treat, and that only the feeblest efforts were exerted to oppose its progress. This was about 1835. In addition, therefore, to encouraging a bold attitude on the part of consumptives to face fresh breezes, even when they were febrile, Dr. Bodington's line of treatment included the administration of a nutritious diet with two glasses of wine every day instead of sub-alimentation on a diet of vegetables, rice and water, and antiphlogistic drugs. One other important measure must be mentioned, as it ranked in the treatment almost equally with the above. This was the giving of "alternate doses of sedatives" during the day, with a full dose at night. The drug preferred was morphine, and this was continued for lengthy periods. It will readily be imagined that patients quickly responded to this new and more rational treatment, especially if they had been subjected already to the effects of the old routine. The diet itself would be much more efficient in reducing the rapidity of the pulse (now ascribed to toxæmia) than the digitalis so frequently given. Dr. Bodington did not lay any special stress on rest out of doors, as is now the custom, but he insisted on carriage exercise for those unable to walk, and he also enunciated the principle of graduated exercise.

It is worth while to examine for a moment the view of the etiology of consumption which Dr. Bodington put forward in his book published in 1840. He came, indeed, astonishingly near the truth in so far as he suggested that the first step in the progress of the disease was the deposition in the substance of the lungs of "tuberculous matter as a foreign body." By this the nervous filaments spread out on the organ were affected so as to lose their power of controlling in healthy action the blood-vessels and cellular tissue; whence would follow the phenomena of inflammation, suppuration, and ulceration. Holding these views, the importance ascribed to nerve sedatives by this acute physician is easily explained. But as regards the main point is not our view to-day identical? Is not consumption caused by the deposit of "tuberculous matter as a foreign body in the lungs"? The severe way in which his conclusions and observations were handled in 1840 by his colleagues and by the reviewers discouraged Dr. Bodington from pursuing a research which might have been of the greatest value. In 1857 tardy justice was done to his work, but though he lived till 1882 he scarcely saw the beginnings of the modern development of his ideas, and did not enjoy the recognition of his shrewd observations and foresight.

